# Fenfluramine treatment in pediatric patients with Dravet syndrome reduces seizure burden and overall healthcare costs: A retrospective and observational real‐world study

**DOI:** 10.1002/epi4.13029

**Published:** 2024-08-14

**Authors:** Cathrine E. Gjerulfsen, Marina Nikanorova, Kern Olofsson, Cecilie Johannessen Landmark, Guido Rubboli, Rikke S. Møller

**Affiliations:** ^1^ Department of Epilepsy Genetics and Personalized Medicine Danish Epilepsy Centre, Filadelfia (Member of ERN EpiCARE) Dianalund Denmark; ^2^ Department of Regional Health Research, Faculty of Health Sciences University of Southern Denmark Odense Denmark; ^3^ Department of Child Neurology Danish Epilepsy Centre Dianalund Denmark; ^4^ Department of Pharmacy Oslo Metropolitan University (Member of the ERN EpiCare) Oslo Norway; ^5^ The National Center for Epilepsy Oslo University Hospital Oslo Norway; ^6^ Department of Pharmacology Oslo University Hospital Oslo Norway; ^7^ Institute of Clinical Medicine University of Copenhagen Copenhagen Denmark

**Keywords:** childhood epilepsy, convulsive seizures, personalized medicine, *SCN1A*

## Abstract

**Objectives:**

Dravet syndrome is a developmental and epileptic encephalopathy characterized by early onset epilepsy with multiple seizure types often intractable to treatment. Randomized clinical trials have demonstrated how treatment with fenfluramine significantly reduces seizure frequency in patients with Dravet syndrome. The study aims to (1) describe the efficacy and tolerability of fenfluramine in a Danish cohort of patients with Dravet syndrome; and (2) evaluate whether treatment with fenfluramine reduces epilepsy‐related hospital contacts administrated by pediatricians or epilepsy‐trained nurses.

**Methods:**

A retrospective registry‐based cohort study at the Danish Epilepsy Centre, Filadelfia, Dianalund, Denmark, enrolled 30 pediatric patients with Dravet syndrome treated with fenfluramine between 2017 and 2023.

**Results:**

Thirty patients with Dravet syndrome (aged 3–21 years, 12 females) with a verified pathogenic *SCN1A* variant were included. They were treated with fenfluramine at a mean duration of 29 months with a mean maintenance dose of 0.5 mg/kg/day. The number of patient‐years on treatment was 75 years. At last follow‐up, 6 patients had discontinued treatment due to lack of efficacy or adverse effects. In the remaining 24 patients, generalized tonic–clonic seizures were reduced by ≥30% in 83%, by ≥50% in 67%, and by 100% in 25%. Additionally, 71% of the patients were reduced in concomitant anti‐seizure medication, and 75% experienced a reduction (mean reduction at 52%, range 11%–94%) in epilepsy‐related hospital contacts from baseline to the end of the treatment period.

**Significance:**

Treatment with fenfluramine effectively reduced seizure frequency and concomitant antiseizure medication in patients with Dravet syndrome. Furthermore, a decrease in epilepsy‐related contacts by 80% was observed over 6 years of treatment, which may indicate cost‐effective benefits.

**Plain Language Summary:**

Patients with Dravet syndrome suffer from severe epileptic seizures that are difficult to treat with medication. Earlier, treatment with fenfluramine (an anti‐seizure medication) has been documented to decrease the total number of seizures in patients with Dravet syndrome. This publication summarizes the experiences with fenfluramine in children with Dravet syndrome at the Danish Epilepsy Centre, Filadelfia, Dianalund, Denmark. Our publication also illustrates that treatment with fenfluramine may reduce the patients' number of yearly contacts with doctors and nurses specialized in epilepsy treatment, which may indicate cost‐effectiveness.

**Key points**

- Our retrospective cohort study enrolled 30 pediatric patients with Dravet syndrome treated with fenfluramine between 2017 and 2023.
- After 1 year of treatment, 100% of the patients continued treatment to study completion, including patients treated for 6 years.
- The majority received sufficient reduction of GTCSs with acceptable tolerability profiles at varying doses.
- From baseline to the end of the treatment period, 71% of the patients were reduced in concomitant ASMs.
- A decrease of 80% in epilepsy‐related contacts with specially trained nurses or treating pediatricians was observed over 6 years of treatment.

## 1 INTRODUCTION

Fenfluramine was initially marketed as an appetite suppressant but withdrawn from the market in 1997 due to associations with cardiac valvulopathy.^1^, ^2^ Based on the results of three phase 3, placebo‐controlled randomized clinical trials (RCTs), demonstrating significantly reduced frequency of major motor seizures in patients with Dravet syndrome (DS), fenfluramine was approved as a repurposed drug for the treatment of DS in 2020.^3^, ^4^, ^5^ Fenfluramine is thought to enhance serotonin (5‐HT) release and serotonergic signaling via different 5‐HT receptors (5‐HT_1D_ and 5‐HT_2A and 2c_) and acts as a positive modulator of sigma‐1 opioid receptors.^6^, ^7^ This will probably affect seizure activity and GABAergic neurotransmission. However, the exact mechanism of action that leads to seizure prevention in patients with DS is not fully clarified.

DS is a developmental and epileptic encephalopathy characterized by early onset intractable epilepsy with multiple seizure types often triggered by elevated body temperature, intellectual disability, behavioral problems, psychiatric comorbidities, and progressive gait impairments.^8^, ^9^, ^10^ DS is primarily caused by loss‐of‐function variants in *SCN1A*, encoding the alpha subunit of the voltage‐gated sodium channel, type 1 (Nav1.1).^11^ Seizures associated with fever begin in the first year of life with subsequent development of afebrile seizure types. The majority of patients with DS experience episodes of status epilepticus; in addition sudden unexpected death in epilepsy (SUDEP) is the main cause of premature mortality.^8^, ^10^ In the Nordic countries, valproic acid is listed as the first‐line treatment for children with DS, while add‐on treatment with stiripentol and clobazam is the second‐line treatment.^8^, ^12^ The treatments used for DS are intended to reduce the seizure burden, and since the majority of patients with DS continue to have poorly controlled seizures, new therapeutic options are necessary.^13^ In international expertise groups, fenfluramine has therefore been suggested as a new second‐line treatment option.^10^ However, data describing the use of fenfluramine in the treatment of seizures in DS in real‐world settings are limited^14^, ^15^, ^16^, ^17^, ^18^ and no “real‐life data” from the treatment of DS with fenfluramine in the Nordic Countries has been reported.

Herein we present data from treatment with fenfluramine of pediatric DS patients in a real‐world setting at the Danish Epilepsy Centre, Filadelfia, Denmark. First, we aim to evaluate the effectiveness and safety profile of fenfluramine in pediatric patients with DS and evaluate if treatment with fenfluramine reduces epilepsy‐related hospital contacts administrated by pediatricians or epilepsy‐trained nurses. Second, we aim to investigate if fenfluramine allows the discontinuation of concomitant anti‐seizure medications (ASMs) in patients with DS.

## 2 METHODS

### 2.1 Study population

We performed a retrospective and observational study of 30 pediatric DS patients treated with fenfluramine at the Danish Epilepsy Centre, Filadelfia, Dianalund, Denmark, between February 2017 and December 2023. Patients aged 2–18 years at the time of fenfluramine treatment initiation were included. All patients harbored a pathogenic *SCN1A* variant. Legal guardians of the patients signed informed consent for participation in this retrospective and non‐interventional study. Further ethics committee review was not required.

### 2.2 Data collection

Data including patient demographics, clinical features, treatment details, and efficacy outcome measures were obtained from electronic medical records as well as seizure diaries compiled by the caregivers. Treatment details included prior and concomitant ASMs, dosage and titration of fenfluramine, length of exposure to fenfluramine, and reported adverse effects. According to guidelines for treatment with fenfluramine, the maximum dose given was 0.7 mg/kg/day with a maximum of 26 mg/day independent of weight in patients not concomitantly treated with stiripentol, and 0.4 mg/kg/day (absolute maximum of 17 mg/day) in patients receiving stiripentol (Fintepla, INN‐fenfluramine (Europa.eu)). Echocardiography was performed every 6 months for the first 2 years and subsequently every year of fenfluramine treatment according to guidelines, and results were obtained from electronic medical records. Demographic information and clinical variables related to epilepsy and treatment were noted before fenfluramine initiation (baseline) and at every year of fenfluramine treatment. The patients were evaluated by neuro‐pediatricians at the Danish Epilepsy Centre every 6th month. All data were entered into a database and de‐identified for patient confidentiality.

Seizure types were defined according to the International League Against Epilepsy.^19^ The primary outcome measure was change in monthly frequency of generalized tonic‐clonic seizures (GTCSs). The average number of GTCSs 3 months prior to fenfluramine initiation determined the baseline frequency of GTCSs. After 1 year of treatment, the frequency of GTCSs was determined based on the mean number of GTCSs in months 11, 12, and 13 of treatment, if a seizure diary was available. Seizure frequency after 2 years of treatment was determined based on the mean number of GTCSs of months 23, 24, and 25. The frequency of GTCSs was determined in the same manner for the years 3, 4, 5, and 6. In the case of absent seizure diaries, the seizure frequency was determined by information in records elaborated by the treating physician. Patients were categorized as “treatment responders” if the frequency of GTCSs was reduced by 30% or more during the treatment period, as this is requested by the Danish Medicines Agency to continue treatment with fenfluramine. Nonresponders to treatment were patients with less than 30% reduction in GTCSs.

The main secondary endpoint was the yearly number of epilepsy‐related hospital contacts. Epilepsy‐related contacts with specially trained nurses or treating pediatricians were counted every year of treatment to quantify a possible positive effect of fenfluramine in a socioeconomic manner. Only one contact per day was taken into consideration. An epilepsy‐related hospital admission was counted as one contact independent of duration. Likewise, adverse effects, adjustments in concomitant ASMs, and reasons for discontinuation of fenfluramine were analyzed.

## 3 RESULTS

### 3.1 Cohort demographics and concomitant treatment

Thirty patients with DS (age 3–21 years, 12 females) were included in the analysis. All habored a pathogenic *SCN1A* variant, of which 14 (47%) were missense and 16 (53%) were protein truncating variants (PTV) (Table 1). Fenfluramine was initiated at a mean age of 9 years (range 2–16 years).

**TABLE 1:** Baseline characteristics of the 30 included pediatric patients with Dravet syndrome treated with fenfluramine.

	*N* = 30
Age at last follow‐up, mean (range)	12 years (4–22 years)
Female, *n* (%)	12 (40%)
Proved SCN1A variant, *n* (%)	30 (100%)
Missense/in‐frame dup. or del., *n* (%)	14 (47%)
PTV, *n* (%)	16 (53%)
Frequency of GTCS at baseline, *n* (%)	
≤5 per month	6 (20%)
<5 and ≥10 per month	10 (33%)
>10 per month	14 (47%)
Concomitant ASMs	
Mean number of ASMs	3
0 ASM, *n* (%)	2 (7%)
1 ASM, *n* (%)	0 (0%)
2 ASMs, *n* (%)	7 (23%)
3 ASMs, *n* (%)	10 (33%)
4 ASMs, *n* (%)	11 (37%)
Most common concomitant ASM	
Valproic acid, *n* (%)	20 (67%)
Stiripentol, *n* (%)	17 (57%)
Clobazam, *n* (%)	15 (50%)
Levetiracetam, *n* (%)	12 (40%)
Additional anti‐seizure therapy	
VNS, *n* (%)	3 (10%)
Ketogenic diet, *n* (%)	1 (3%)

At baseline, 6/30 (20%) patients experienced ≤5 GTCSs per month, whereas 10/30 (33%) had >5 and ≤10 GTCSs per month, and 14/30 (47%) of the patients >10 GTCSs per month. In total, 21/30 (70%) patients were treated with at least 3 concomitant ASMs. The most commonly prescribed ASMs were valproic acid (20/30), stiripentol (17/30), and clobazam (15/30). At baseline, 1/30 (3%) were treated with ketogenic diet and 3/30 (10%) had a Vagus Nerve Stimulator (VNS).

### 3.2 High retention with a mean maintenance dose of 0.5 mg/kg/day

**FIGURE 1:**
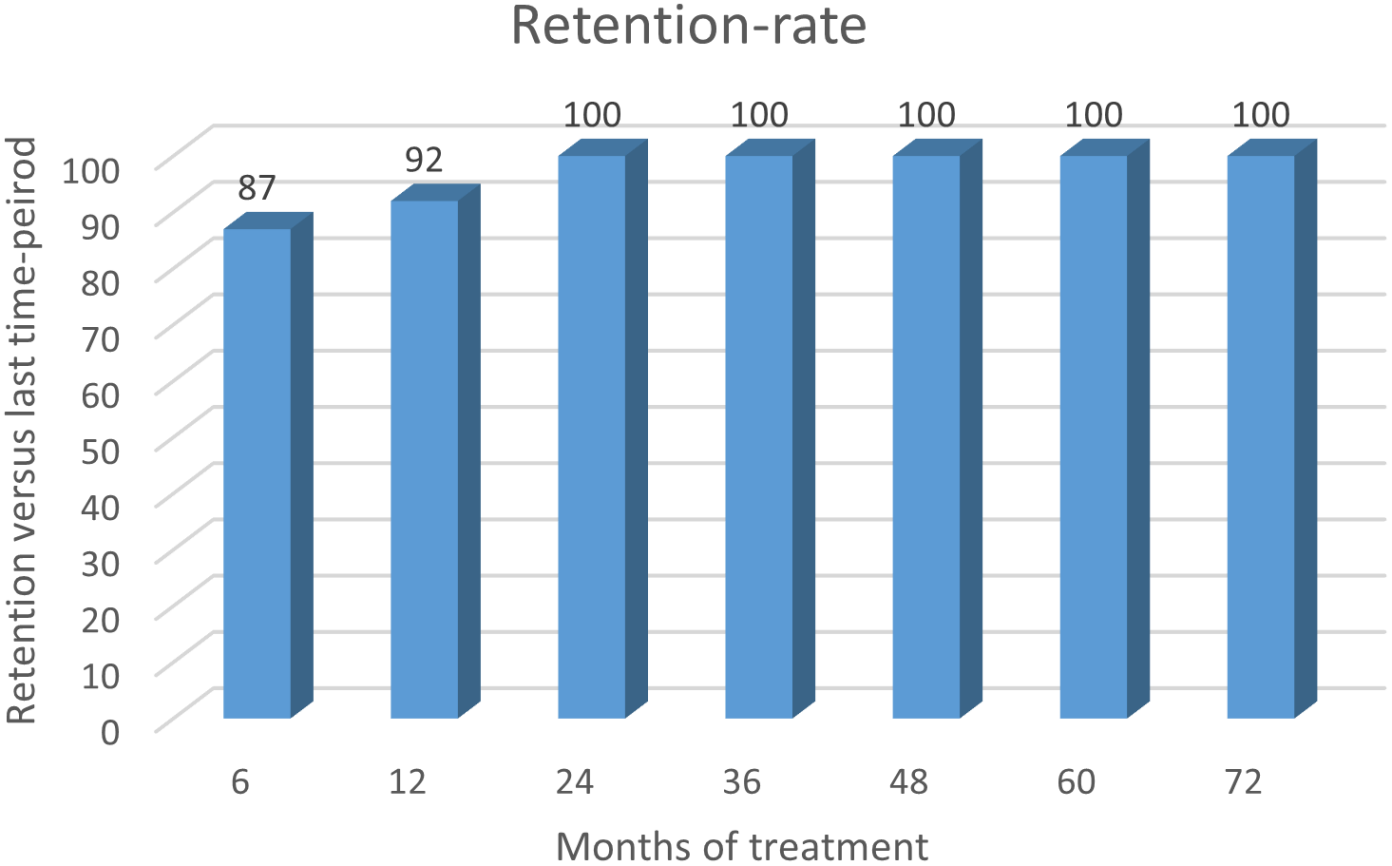
The cohort's retention rate was high, as 87% of patients in treatment with fenfluramine continued treatment >6 months. After 1 year of treatment, 100% of the patients continued treatment to study completion.

To evaluate the retention rate and potential termination of treatment, each patient was followed until the end of the study period in December 2023. During the first 6 months of fenfluramine administration, 4 patients discontinued fenfluramine. In the following 6 months, additionally, 2 patients stopped their treatment. Hence in our cohort, 87% of patients in treatment with fenfluramine continued their treatment >6 months, and 92% of the patients still receiving treatment at 6 months continued at least until the end of year 1. After 1 year of treatment, 100% of the patients continued treatment to study completion, which includes patients that have been treated for 6 years (Figure 1).

The main reason for the discontinuation of fenfluramine was adverse events. None of the patients who terminated treatment reached the recommended maximum dose of fenfluramine (0.7 mg/kg/day or 26 mg/day). The 80% of the patients who continued fenfluramine received sufficient reduction of their seizures with acceptable tolerability profile at varying doses. Of the 24 patients, who continued treatment, 11 patients (46%) received the maximum maintenance dose of fenfluramine (0.7 mg/kg/day (26 mg/day)), while 5 patients (21%) received a maintenance dose at <0.7 and ≥0.5 mg/kg/day, and the remaining 8 patients (33%) a maintenance dose at <0.5 mg/kg/day. At last follow‐up, only 3/24 (12.5%) patients were concomitantly treated with stiripentol. The mean dose in the whole cohort of patients treated for >12 months was 0.5 mg/kg/day.

### 3.3 Seizure reduction by fenfluramine

In total, 6/24 patients (25%, 20% of the total cohort) were free of GTCSs at the end of the study period. These patients all initiated treatment with fenfluramine in 2017; three became seizure‐free between 6 and 12 months of treatment, and the remaining three patients became free of GTCS after two (2) and three (1) years of treatment, respectively.

**FIGURE 2:**
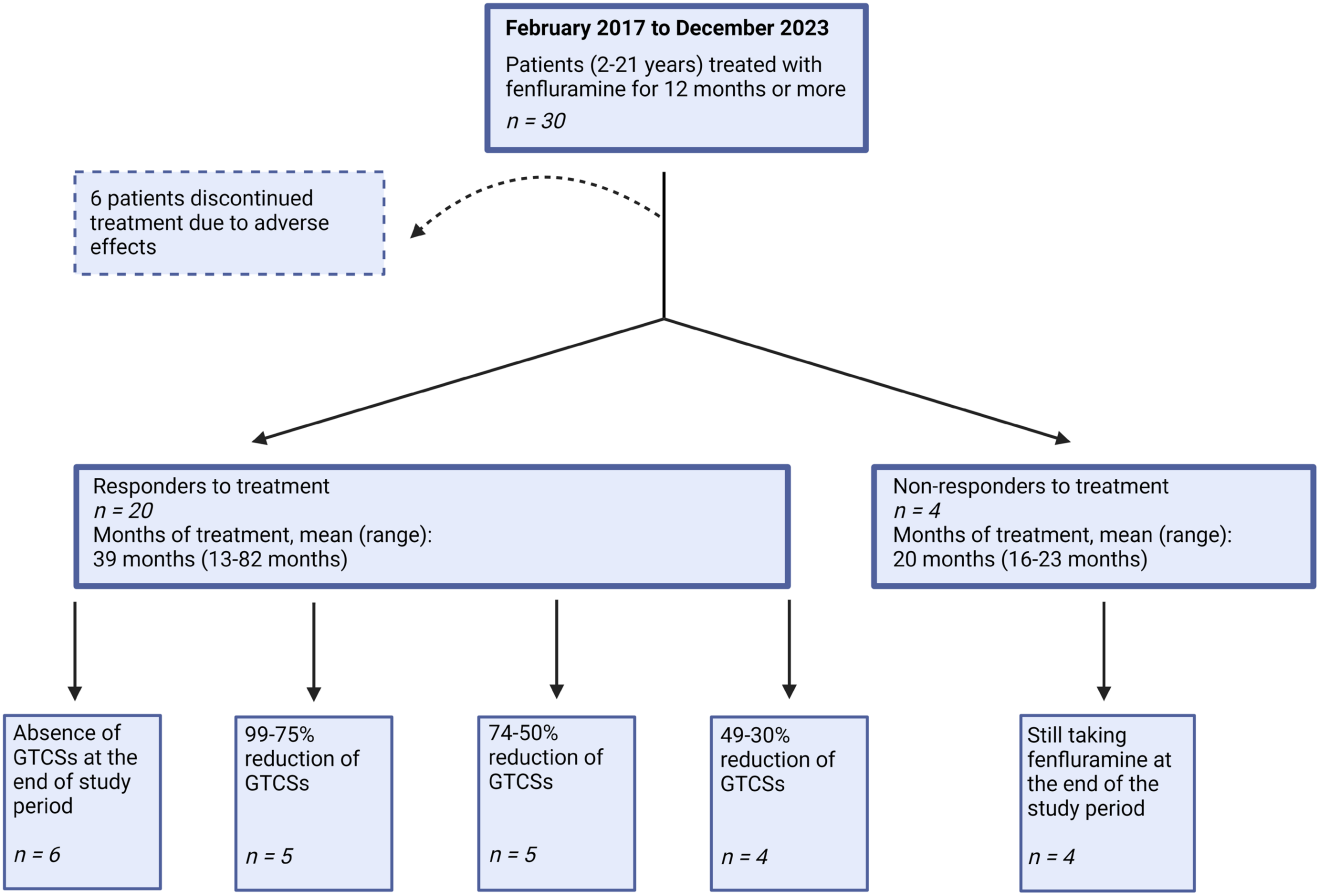
Overview of the retention and reduction of generalized tonic‐clonic seizures (GTCSs). Responders to fenfluramine treatment achieved a reduction of GTCSs by 30% or more. The figure is created with BioRender.com.

In 5/24 patients (21%, 17% of the total cohort), treatment with fenfluramine led to a 99–75% reduction in frequency of GTCSs, while another 5/24 (21%) experienced a 74–50% reduction (Figure 2). In 4/24 patients (17%, 13% of the total cohort) the frequency of GTCSs was reduced by 49–30%. Thus, 16/24 patients (67%, 53% of the total cohort) achieved a seizure reduction at ≥50%. The remaining 4/24 patients (17%) had a reduction in frequency of GTCSs of less than 30%, but continued treatment due to other beneficial effects of fenfluramine; two patients experienced less frequent status epilepticus episodes, but a reduction in GTCS was not identified. Another patient defined as a non‐responder experienced GTCSs with shorter duration and less intensity. Additionally, one patient had a reduction of GTCSs of 29%, and continued the treatment due to the positive effect, although it was lower than 30%.

Eleven of 24 (46%) patients received the maximum dose of 0.7 mg/kg/day or 26 mg/kg/day. Of these 11 patients, 5 experienced a reduction of GTCSs greater than 75%, while 3 experienced a 74–50% reduction, and 3 experienced a reduction less than 49% (Table 2). The lowest mean age at initiation of fenfluramine treatment was found in the group of patients who had a 74–50% reduction of GTCSs, while the highest mean age was observed in the group of responders with a 49–30% reduction. Patients were divided into three groups defined by the frequency of GTCSs at baseline (Table 2). A trend regarding seizure reduction and baseline frequency of GTCSs was not observed.

**TABLE 2:** Treatment retention, fenfluramine dose, and seizure outcomes in the 24 Dravet patients, that continued treatment with fenfluramine.

	Absence of GTCSs	99–75% reduction of GTCSs	74–50% reduction of GTCSs	49–30% reduction of GTCSs	No change in the frequency of GTCSs
Total cohort (*n* = 24), (%)	6 (25%)	5 (21%)	5 (21%)	4 (17%)	4 (17%)
Age at fenfluramine initiation, mean (range)	9.5 years (5–15 years)	8.5 years (4–16 years)	6.8 years (3–10 years)	10 (7–14 years)	8 years (2–16 years)
Length of treatment, whole years (%)					
≥1 and <3 years, (*n* = 16)	1/16 (6%)	4/16 (25%)	5/16 (31%)	2/16 (13%)	4/16 (25%)
≥3 years and up, (*n* = 8)	5/8 (62%)	1/8 (13%)	0 (0%)	2/8 (25%)	0 (0%)
Frequency of GTCSs at baseline					
≤5 per month (*n* = 6)	3/6 (50%)	0	2/6 (33%)	0	1/6 (17%)
>5 and ≤10 per month (*n* = 8)	0	4/8 (50%)	2/8 (25%)	1/8 (12.5%)	1/8 (12.5%)
>10 per month (*n* = 9)	3/9 (33%)	1/9 (11%)	0	3/9 (33%)	2/9 (22%)
Fenfluramine maintenance dose					
0.7 mg/kg/day, maximum 26 mg/day, (*n* = 11)	5/11 (45%)	0	3/11 (27%)	1/11 (9%)	2/11 (18%)
<0.7 mg/kg/day and ≥0.5 mg/kg/day, (*n* = 5)	0	2/5 (40%)	2/5 (40%)	0	1/5 (20%)
<0.5 mg/kg/day, (*n* = 8)	2/8 (25%)	3/8 (38%)	0	2/8 (25%)	1/8 (12%)
Concomitant treatment with stiripentol, (*n* = 3/8)	(0)	(2/3) (66%)	(0)	(1/3) (33%)	(0)

### 3.4 Fenfluramine reduces epilepsy‐related contacts with healthcare and the burden of concomitant ASMs

**FIGURE 3:**
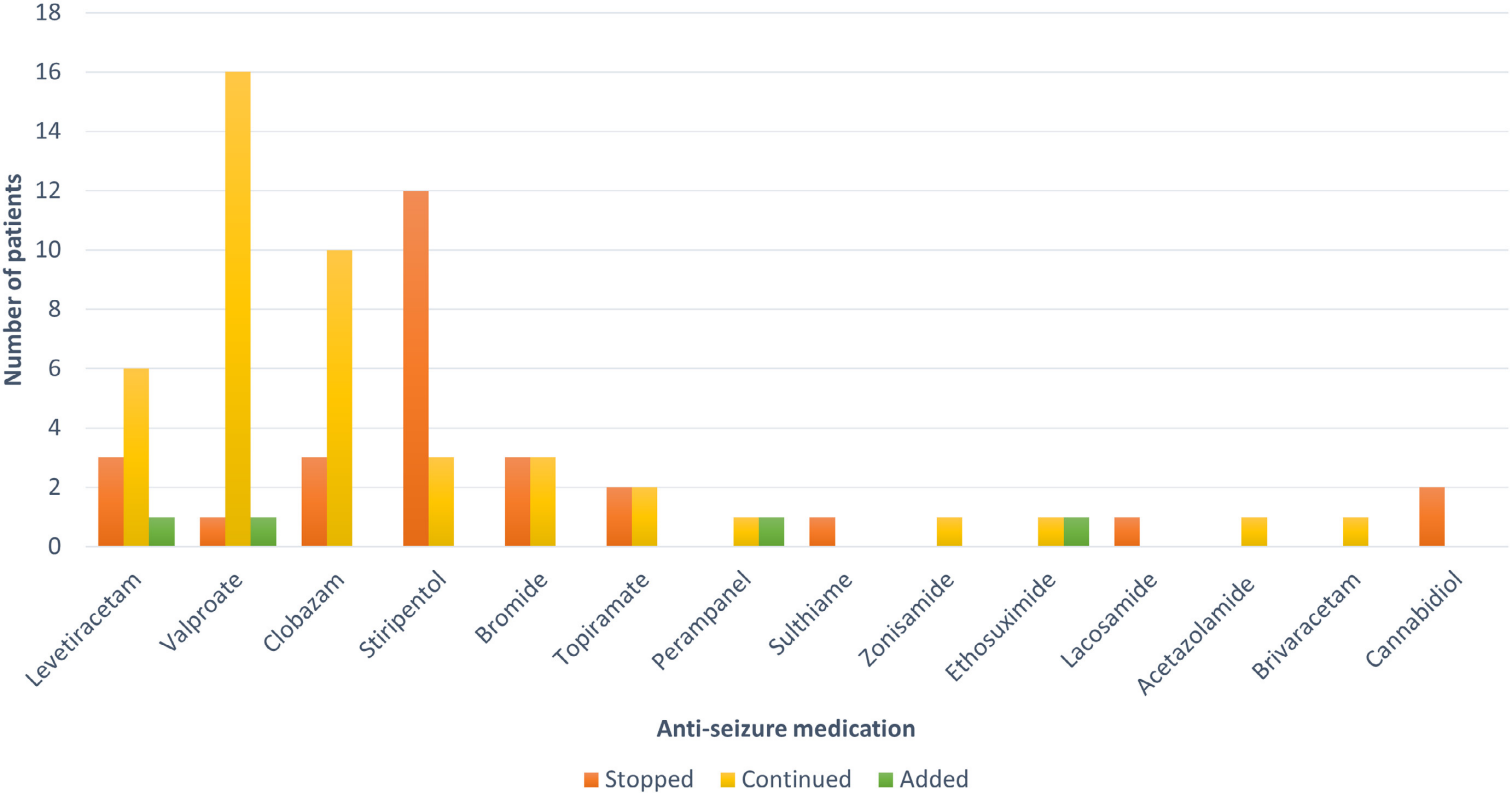
Changes in concomitant anti‐seizure medication (ASMs) from baseline to the end of the treatment period. The registered changes include ASMs that have been added, discontinued, or changed doses during the treatment period.

From baseline to the end of the treatment period, 17/24 (71%) were reduced in concomitant ASMs. The ASM most frequently discontinued was stiripentol, while most patients typically continued treatment with valproic acid and clobazam (Figure 3). Of the 24 patients who continued treatment, 15 received stiripentol prior to treatment with fenfluramine, and 12 (80%) patients discontinued stiripentol after fenfluramine was initiated. Only 4/24 (17%) patients had another ASM added after treatment with fenfluramine was initiated.

**FIGURE 4:**
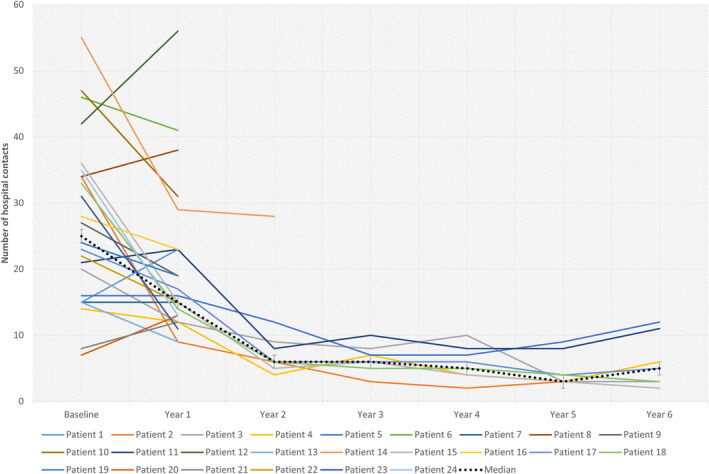
Number of epilepsy‐related contacts to pediatricians or special‐trained nurses counted every year of treatment with fenfluramine.

Figure 4 illustrates the number of epilepsy‐related contacts to pediatricians and special‐trained nurses counted every year of fenfluramine treatment. The vast majority of the patients (18/24, 75%) had a reduction (mean reduction at 52%, range 11–94%) in hospital contacts from baseline to the end of the treatment period. One patient had the same number of hospital contacts whereas five patients experienced a small increase in epilepsy‐related hospital contacts after the treatment was initiated (mean increase at 30%, range 11–46%).

### 3.5 Safety and tolerability

During the treatment period, 16/30 (53%) patients reported fenfluramine‐related adverse effects consisting of impaired balance (7/16 patients), reduced appetite (6/16 patients), increased seizure frequency (5/16 patients), tiredness/somnolence (2/16), and tremor (2/16 patients). The reported adverse effects were temporary in 10/16 of the patients and no patients reported adverse effects 1 year after the therapy with fenfluramine was initiated. Of the 30 patients, 6 discontinued treatment with fenfluramine; 2/6 due to increased seizure frequency, 3/6 due to a combination of adverse effects (loss of appetite, impaired balance, aggressive behavior, and incontinence) and increased seizure frequency, and one patient stopped treatment with fenfluramine due to adverse effects (loss of appetite and impaired balance) and low efficacy.

Echocardiography was performed every 6 months for the first 2 years and subsequently every year of fenfluramine treatment, and no abnormalities were observed.

## 4 DISCUSSION

Compilation of retrospective real‐world data on new ASMs may contribute to collecting further information about efficacy and safety not found in RCTs. In this study, we report our experience with the use of fenfluramine in a pediatric cohort of 30 patients with DS followed at the Danish Epilepsy Centre, Dianalund, Denmark. We aim to test the effectiveness of fenfluramine in the cohort and evaluate whether the treatment may be cost‐effective. This is measured by epilepsy‐related hospital contacts counted for every year of treatment. Second, we aim to investigate if fenfluramine treatment allows the discontinuation of concomitant ASMs and evaluate doses of fenfluramine and the safety profile in general.

The Danish Medicines Agency demands a seizure reduction at a minimum of 30% to continue treatment with fenfluramine, which in this study was defined as “responders.” Among the responders (20 patients), we found a mean reduction of GTCSs of 60% over a mean treatment period of 39 months, whereas 75% had a reduction in GTCSs of ≥50%. Among these 75%, six patients (20% of the total cohort) reported a reduction in GTCSs at 100% at the end of the treatment period. This correlates with real‐world data published in previous studies, in which 54–69% achieved a reduction in GTCSs of ≥50%, and 12–17% achieved seizure freedom regarding GTCSs.^17^, ^18^ Nonresponders were treated with fenfluramine for a mean period of 20 months and reported a mean reduction of GTCSs at 9%. However, they all continued treatment with fenfluramine due to other beneficial and meaningful outcomes (GTCSs of shorter duration, and less frequent status epilepticus), which is of great importance when evaluating the response to treatment.

Epilepsy‐related contacts with nurses or pediatricians were counted every year of treatment with fenfluramine as a novel approach to elucidate cost‐effectiveness in patients with DS. To our knowledge, this is the first publication reporting the development of epilepsy‐related contacts with hospitals when treated with fenfluramine. Patients with DS require highly specialized healthcare due to high seizure frequency, emergency visits, frequent status epilepticus, and several comorbidities. Seizure‐related costs contribute to a large proportion of the estimated all‐course medical costs, which for a patient with DS are 12 times greater than for age‐matched members of the general population.^20^ Naturally, insufficient seizure control increases seizure‐related costs, which makes adequate treatment of seizures crucial to the patient and society. From baseline to the end of the observed treatment period, 75% of the patients experienced a mean reduction of 52% in epilepsy‐related hospital contacts. From baseline, the median number of epilepsy‐related contacts was reduced from 25 to 6 contacts per treated patient per year (a reduction of 75%) after 2 years of treatment. Over 6 years, the median number of epilepsy‐related hospital contacts was reduced by 80% per treated patient. This indirectly proves the effectiveness of fenfluramine treatment and indicates cost‐effective benefits. It is known, that the seizure frequency in patients with DS often stabilizes over time, and status epilepticus gets less frequent through the years especially when patients reach adulthood.^21^ This may lead to a natural reduction in epilepsy‐related hospital contacts which could influence our results. However, a reduction of 75% was observed already after 2 years of treatment with fenfluramine, which in combination with the mean age of our cohort at 12 years, makes it most likely, that the reduction is due to treatment with fenfluramine and not age‐dependent seizure reduction.

Five patients experienced an increase in hospital contacts after initiation of fenfluramine treatment. Of these, three were responders with an average reduction in GTCSs at 61% (57–71%). As the Danish Epilepsy Centre is the only place in Denmark allowed to dispense fenfluramine, the increase in hospital contacts experienced by two out of these three patients may be due to the referral of the patients to the Danish Epilepsy Centre. These two patients were then followed at two hospitals which may have increased the number of epilepsy‐related contacts. The other responders with increased epilepsy‐related hospital contacts were monitored closely due to increased seizure frequency when stiripentol was tapered off. The remaining two patients did not respond to treatment (reduction of GTCSs at 0% and 7%), and one of them had a VNS implanted during the treatment period with fenfluramine, which increased the number of epilepsy‐related hospital contacts.

From baseline to the end of the treatment period, 17/24 responders (57% of the total cohort) were reduced in the total number of concomitant ASMs, and stiripentol was the most frequently discontinued drug. This is in the order of 12–28% higher than what previously has been found in real‐life observations.^15^, ^17^, ^18^, ^22^ As some of the ASMs used to reduce seizure burden in patients with DS may contribute to cognitive dysfunction,^12^, ^23^ the decrease in ASM consumption may positively influence the patients' long‐term cognitive abilities, quality of life in general, and reduce the risks associated to polytherapy (e.g., drug–drug interactions and adverse effects). An approach including more personalized therapies in the treatment of rare diseases may be more efficient.^24^ In our cohort of 24 patients who remained on fenfluramine, four patients had one ASM substituted for another after fenfluramine was initiated. Due to the replacement, the overall number of concomitant ASMs was not increased.

In previously performed RCTs, a trend regarding the dose of fenfluramine and response was observed as fenfluramine 0.7 mg/kg/day was associated with higher seizure reduction.^3^ This trend regarding seizure response and fenfluramine dose has not been identified in our study, as the response distribution of patients receiving a dose less than 0.7 mg/kg/day (absolute maximum of 26 mg/day) resembles the one with patients reaching maximum dose. In contrast to the RCTs, three of our patients receiving a low dose of fenfluramine were treated concomitantly with stiripentol which interacts with fenfluramine. As fenfluramine is a substrate of some of the CYP450 enzymes that stiripentol may inhibit (CYP2C19, 3A4, and 2D6), the combination of stiripentol and fenfluramine may require a dose reduction of fenfluramine,^25^, ^26^ which result in a lower maintenance dose. In our cohort, the combination of fenfluramine and stiripentol was not associated with a higher reduction of seizures, which correlates with previously published real‐world data.^15^, ^18^, ^22^ An association between discontinuation of fenfluramine and treatment with stiripentol and fenfluramine in a combination was not identified in our cohort either, as only two of the six patients, who discontinued fenfluramine, received both drugs. Neither age at fenfluramine initiation nor the frequency of GTCSs at baseline seemed to influence the response to fenfluramine, which indicates that treatment with fenfluramine may benefit patients with diverse severity and in different age groups.

In our cohort, the most frequently observed adverse effects included impaired balance, reduced appetite, increased seizure frequency, tiredness/somnolence, and tremor. These adverse effects corresponded to the ones reported in the RCT studies^3^, ^4^, ^5^ and the reported use of fenfluramine in real‐world settings,^15^, ^17^, ^18^, ^22^ except for the occurrence of impaired balance. However, the tendency to fall is a known adverse effect of fenfluramine and may be related to somnolence, which also was observed in our cohort. The influence of fenfluramine treatment on epilepsy‐related contacts with health services has not previously been reported. Our results may indicate a cost‐effective benefit of fenfluramine in the treatment of patients with DS. Together with improvements in quality of life,^27^ lower risk of SUDEP,^28^ and a clinically meaningful improvement in executive function in patients with DS treated with fenfluramine,^29^ a reduction in hospital contacts provides insights in the beneficial socio‐economic value of fenfluramine treatment.

### 4.1 Methodological aspects

In this study, a patient group with a rare genetic epilepsy syndrome has been treated with a novel repurposed drug with an extensive follow‐up time of up to 6 years. The study has some limitations worth taking into account. First, as a retrospective and non‐interventional study, the treatment with fenfluramine was neither randomized nor blinded to patients or physicians. Second, only GTCSs were counted, as these seizures are valid data reported from caregivers, and associated with a higher risk of SUDEP. An attempt to measure cost‐effectiveness was performed by counting the number of epilepsy‐related contacts over time, which is a new approach in such settings. This study did not, however, include non‐seizure‐related outcomes such as quality of life, clinical global impression, cognitive performance, etc. Studies designed for detecting non‐seizure‐related improvements due to fenfluramine treatment are needed and are planned to be conducted by this group in the future to further expand the knowledge of treatment with fenfluramine in patients with DS. The small sample size is also a limitation of the study. However, DS is a rare disease, and not all pediatric patients with DS in Denmark can be treated with fenfluramine due to current Danish guidelines requirements. In our study, we only included pediatric patients. The inclusion of adult patients may further increase our knowledge about the treatment with fenfluramine in patients with DS. To further evaluate the pharmacokinetic aspects and drug interactions of fenfluramine treatment serum concentrations measurements will be implemented in future evaluation, as these patients require close follow‐up and monitoring.^30^

In conclusion, two‐thirds of the patients in our cohort experienced a meaningful reduction in GTCSs after add‐on therapy with fenfluramine, including six patients with a 100% reduction of GTCSs in a period of up to 6 years. Additionally, long‐term (6‐years) treatment with fenfluramine showed a reduction of up to 80% in epilepsy‐related hospital contacts. In our cohort, 71% of the patients discontinued stiripentol, and 71% reduced the overall burden of concomitant ASMs. Our study also confirmed fenfluramine as a safe and well‐tolerated treatment, as no abnormalities at echocardiography were observed, and no patients reported adverse effects one year after treatment was initiated.

## FUNDING INFORMATION

The first author received funding from UCB Pharma. The donator did not influence data collection and analysis as well as manuscript writing.

## CONFLICT OF INTEREST STATEMENT

CJL has received speaker honoraria from Angelini, Eisai, Jazz, and UCB Pharma. RSM has received consulting fees from UCB, Orion, Saniona and Immedica, and speaker fees from EISAI, Angelini Pharma, Jazz Pharmaceuticals, Orion and UCB. The remaining authors have no conflict of interest. We confirm that we have read the Journal's position on issues involved in ethical publication and affirm that this report is consistent with those guidelines.

## Data Availability

The data that support the findings of this study are available on request from the corresponding author. The data are not publicly available due to privacy or ethical restrictions.
